# How Posture and Previous Sensorimotor Experience Influence Muscle Activity during Gait Imagery in Young Healthy Individuals

**DOI:** 10.3390/brainsci13111605

**Published:** 2023-11-19

**Authors:** Barbora Kolářová, Marek Tomsa, Petr Kolář, Hana Haltmar, Tereza Diatelová, Miroslav Janura

**Affiliations:** 1 Department of Clinical Rehabilitation, Faculty of Health Sciences, Palacký University Olomouc, Hněvotínská 976/3, 775 15 Olomouc, Czech Republic; marek.tomsa@fnol.cz (M.T.); petr.kolar@fnol.cz (P.K.);; 2 Department of Rehabilitation, University Hospital Olomouc, Zdravotníků 248/7, 779 00 Olomouc, Czech Republic; 3 Department of Natural Sciences in Kinanthropology, Faculty of Physical Culture, Palacký University Olomouc, třída Míru 117, 771 11 Olomouc, Czech Republic; miroslav.janura@upol.cz

**Keywords:** motor imagery, gait, muscle activity, surface electromyography

## Abstract

This study explores how gait imagery (GI) influences lower-limb muscle activity with respect to posture and previous walking experience. We utilized surface electromyography (sEMG) in 36 healthy young individuals aged 24 (±1.1) years to identify muscle activity during a non-gait imagery task (non-GI), as well as GI tasks before (GI-1) and after the execution of walking (GI-2), with assessments performed in both sitting and standing postures. The sEMG was recorded on both lower limbs on the tibialis anterior (TA) and on the gastrocnemius medialis (GM) for all tested tasks. As a result, a significant muscle activity decrease was found in the right TA for GI-1 compared to GI-2 in both sitting (*p* = 0.008) and standing (*p* = 0.01) positions. In the left TA, the activity decreased in the sitting posture during non-GI (*p* = 0.004) and GI-1 (*p* = 0.009) in comparison to GI-2. No differences were found for GM. The subjective level of imagination difficulty improved for GI-2 in comparison to GI-1 in both postures (*p <* 0.001). Previous sensorimotor experience with real gait execution and sitting posture potentiate TA activity decrease during GI. These findings contribute to the understanding of neural mechanisms beyond GI.

## 1. Introduction

Motor imagery is defined as a purely cognitive process where the individual imagines making a movement and the imagery is not accompanied by any visible manifestation of such a movement. Motor imagery is an active process by which the representation of a certain action is internally reproduced via motor memory, while the movement itself is inhibited and the somatosensory inputs generated during motion are absent [1,2,3,4]. Within the motor imagery, the brain creates a so-called internal kinaesthetic model of the movement [1,5], which has potential in motor learning processes [5,6,7]. A proper understating of the neural correlates behind motor imagery is valuable beyond its capacity to deepen knowledge concerning gait control mechanisms [6,8] and can be used to further investigate sensorimotor adaptations resulting from motor imagery training and [6,7] to improve rehabilitation settings via the utilization of gait imagery as part of therapy to potentiate motor recovery [8,9,10,11].

Motor imagery manifests similar patterns of neural activity to actual movements [9,12,13,14]. Gait imagery (GI) actually represents the paradigm for studying brain activity during walking as it serves as a proxy for motor execution [14,15,16] in both healthy subjects [6,7] and in patients with neurological disorders, such as patients with Parkinson’s disease or post-stroke patients [10,17].

Motor imagery shares the activity of specific neural substrates similarly to during movement execution [3,4,14,18,19,20]. The activity of the brain cortical and subcortical structures directly responsible for motor control have been proven to increase when imagining a movement [4,7,12,13,14,21]. A meta-analysis conducted by Hétu [14] revealed that the motor imagery of lower-limb movements, including gait, rely mainly on the parietal regions, supplementary motor area, cerebellum and putamen. It has been suggested that the activity of these areas is more necessary for gait programming than for real stereotype locomotion, which is more automatic [14,22,23,24,25]. Cortical centers become engaged to a greater extent when the demands of a locomotor task require increasing cognitive or sensory information processing [7]. On the basis of the assumption that motor execution and motor imagery share similar neural structures [6,14,19], imagined locomotion has been proposed as a paradigm used to study brain activation during walking using functional magnetic resonance imaging [14,26].

Even though the brain activity that is connected to motion imagination has received quite a lot of attention, studies focusing on the changes in the excitability on the spinal level [18,27,28] or changes in muscle activity [18,26,29] are not that widespread. Several studies proved that motor imagery increases excitability in the corticospinal tract, which projects to the motoneurons and interneurons controlling the target muscles [20,28,30,31]. Also, the excitability of spinal reflexes or muscle spindle Ia afferent fibers was proved to increase [18], which may also influence muscle activation.

From the motor control perspective, the influence of motor imagery on muscle activity may represent the missing piece of evidence behind the more complex effect of motor imagery as the motoneuron pools of muscles involved in imagined movements receive summations of inputs from both descending neural pathways and from sensory afferents, similarly to motion execution.

The fact that movement imagery influences muscle activity has been unambiguously proven by studies pointing to the increased muscle power resulting only from mental training [32] and the positive effect of motor imagery during either inter-trial recovery periods on maximal isometric force [33]. The influence of motor imagery on electromyographic activity has previously been investigated, mostly for the upper limbs [29]. Nevertheless, the presence and characteristics of the surface electromyographic (sEMG) signals detected when imagining a movement are still relatively inconsistent. In some cases, the results of studies conducted on upper-limb functional tasks proved that sEMG activity increased during motor imagery in comparison with the non-imagery condition [29,34]. The same was proved for some non-gait lower-limb tasks [18,26]. Some studies concerning lower-limb muscle activity during motion imagination highlighted that motor imagery did not lead to changes in muscle activity when imagining, e.g., walking up-stairs [35] or standing on tiptoes [36]. In relation to GI, there was proven to be either no influence, or an inhibitory influence, on the activity of lower-limb muscles [7,37]. This inhibitory effect was proved already for the distal leg muscles tibialis anterior and gastrocnemius medialis during gait imagination paced externally by a metronome [37]. However, what is true is that the motor control mechanisms of lower-limb motion (including gait) rely on the use of different cerebral and spinal networks compared to upper-limb movements, and real gait execution without any extra demands is more or less automatic and thus is less dependent on central motor commands [14,38]. This is probably mostly true for distal leg muscles during gait [37].

Human gait is considered a complex motor task that involves coordinated muscle activation and balance control, as well as the adaptation of movements to the external environment. The rhythmic complex patterns of the muscle activity required for locomotion are, to a great extent, driven by the neural networks at the spinal level, referred to as central pattern generators (CPGs) [14,22,23,24,25]. The activity of the spinal locomotor CPGs is regulated by supraspinal structures and modulated by afferent feedback from the periphery [14,23,24,26,38,39]. The supraspinal control of CPGs is realized via the mesencephalic locomotor region and brainstem structures [40], whose activity is dependent, to a great extent, upon subcortical and cortical inputs in humans, particularly in the context of the programming of movement, considering the changes in demands and with respect to the external environment [38]. Therefore, the question is whether we should expect similar results for muscle activity behavior for gait imagery tasks as for upper-limb tasks. It also remains unclear how afferent sensory information from the periphery may contribute to potential sEMG activation when imagining gait [34].

Based on the published studies [27,30], it seems that muscle activity, when imagining movement, is modulated not only by the imagery itself but also by other factors. For example, it appears that the effect of movement imagination on neural structures is more pronounced within complex motor tasks in comparison to simple ones [20]. Also, factors such as the character of the afferent somatosensory feedback from the periphery deserve attention. For instance, a greater modulation in muscle activity occurs in the situation where the movement imagery is accompanied by actual sensory feedback [9,29,31,41,42] or where the initial posture during imagery is identical to the initial posture for realization of the movement [30,31,42]. The issue of the initial posture when imagining movement ranks among the actual topics [43], owing to the potential relevance of the reflexive modulation of muscle activity [22,39,44,45,46]. Posture, which is congruent with imagined motion, provides more appropriate somatosensory feedback and thus may be used to develop a more accurate predictive internal model of motion [47] for further motion execution.

The question arising from this topic is whether sitting (posture incongruent with walking) or standing (posture congruent with walking) influences the final electromyographic outcome during GI as both these positions provide different somatosensory feedback. The aim of the study was to assess how default sitting or standing posture and immediate previous sensorimotor experience with real walking modulate activity of distal lower-limb muscles during gait imagery in young, healthy individuals.

## 2. Materials and Methods

### 2.1. Participants

The study involved 36 young healthy individuals (23 women and 13 men). The average age of the participants was 24 (±1.1) years, with a height of 164 (±8.9) cm and a weight of 68 (±12.3) kg. All participants who volunteered to participate in this study were recruited from university students of a master’s program in Physiotherapy. All participants partook in leisure sport activities and none of them were sport professionals. All participants provided informed consent. The research was approved by the local ethics committee (Ethics Committee of the Faculty of Health Sciences, Palacky University Olomouc with approval code UPOL-2945/1040-2018). All procedures were performed according to the ethical standards of the Declaration of Helsinki.

The criterion for participation in the study was good motor imagery—classified as being at least mark 3 on average, in accordance with the standardized Movement Imagery Questionnaire—Revised (MIQ-R). Therefore, only participants with at least moderate imagery ability were recruited. MIQ-R, which represents a valid and reliable instrument for measuring motor imagery ability in healthy persons [48,49], uses 7-point scales (from 1 for ”very hard to see or feel ” to 7 for “very easy to see or feel”) within an 8-item self-report questionnaire [48,49].

Another criterion for participation in the study was the absence of any acute postinjury condition, a neurological condition, an orthopedic condition, pain, or a cognitive deficit that could in any way restrict or preclude the measurement.

### 2.2. Instrumentation

The influence of GI on muscle activity was assessed through telemetric sEMG using hybrid bipolar electrodes with default inter-electrode distance (Delsys Trigno, Natick, MA, USA) in sitting and standing postures.

Muscle activity was recorded from the tibialis anterior (TA) and gastrocnemius medialis (GM) in both lower limbs. The electrodes were placed onto the belly of the respective muscles with self-adhesive straps; then, the palpation of muscle bellies was conducted with sub-maximal isometric contractions according to the standards set by SENIAM [50]. Prior to the application of electrodes, the skin over the muscle bellies was shaved and cleaned using abrasive paste to reduce skin impedance. Muscle activity was recorded for all situations tested within the experimental protocol.

### 2.3. Experiment Protocol

**Figure 1:**
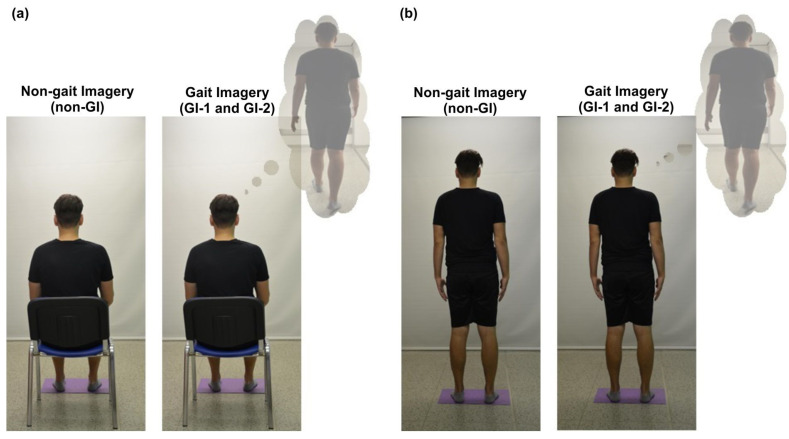
Experimental conditions in the standardized sitting (**a**) and standing (**b**) positions.

The initial postures for all tested situations and participants were always sitting or standing with eyes open, facing the white screen (see Figure 1). The position of the participants’ feet was normalized for both postures with respect to pelvic width and with ankles aligned with the line. To ensure feet were in an identical position for all tested situations, the initial feet position was drawn on paper and the tested participant always stood on this template during the measurement. In the sitting posture (with the seat height 45 cm), the participant sat upright with no support for the back and upper limbs, which were laid freely on the individual’s thighs. In the standing posture, the participant stood upright, with the upper limbs hanging freely along the body. To exclude the possible influence of the order of the initial positions on the results of the measurement, their order was randomized. In both sitting and standing postures, muscle activity was measured for the following 3 test situations:

1. In an initial posture without imagining any movement (non-GI). To prevent the generation of any other imagery that would be undesirable during this task, the participants imagined singing the song “Happy Birthday”. The participants were asked by the researcher to sing the song in their mind and, after 30 s, were given a signal to stop.

2. In an identical position, the participants were instructed to imagine walking along a corridor (gait imagery before walking (GI-1)) as precisely as possible. Prior to the imagery, all participants saw the corridor, which was outside a laboratory. The instructions given to every participant before the measurement began were as follows: “Imagine yourself walking the corridor you have just seen at your comfortable gait speed”. After 30 s, the participants were given a signal to stop.

The participants were subsequently asked to walk at their own comfortable speed along the corridor outside a laboratory.

3. The participants took the same initial position as in situations 1 and 2. They were instructed to imagine walking the corridor they had just walked (gait imagery after walking (GI-2)). The instructions were as follows: “Imagine yourself walking the corridor you have just walked at your comfortable speed”. After 30 s, the participants were given a signal to stop.

The time duration of 30 s of imagination reflected the self-reported optimal time duration of stereotype gait imagination.

Electromyographic activity was recorded throughout all 3 experimental situations, which are illustratively demonstrated in Figure 1.

### 2.4. Subjective Assessment of GI

For situations 2 and 3 (i.e., situations where participants imagined gait), every participant was asked to write the subjectively perceived level of simplicity/difficulty of GI on a four-point scale, where classification 1 referred to significant difficulty, 2 to moderate difficulty, 3 to moderate simplicity and 4 to significant simplicity in imagining the gait.

All testing procedures were realized in a quiet room in one day.

### 2.5. Data Processing

The processing of electromyographic data was performed in EMGworks^®^ Analysis software (version 4.1.7, Delsys Trigno, Natick, MA, USA), where the 10 s in the middle of the record, without artefacts, was evaluated. Data were processed by removing the mean value and half waves were rectified using the root-mean-square algorithm (with a window size of 0.25 and a window overlap of 0.05 s) and were high-pass filtered at 20 Hz. Data were subsequently used to calculate the mean values of muscle activity (EMGrms) for all tested muscles and for all three tested conditions in both the sitting and standing postures. These mean values were statistically analyzed.

### 2.6. Data Analysis

The statistical processing of data was performed using STATISTICA software (version 13.4.0). The differences in muscle activity for the individually tested situations were assessed using Friedman’s ANOVA test. Regarding the non-normal data distribution, the method selected for within-group comparison and the post hoc test was the Wilcoxon test for two paired samples, with Bonferroni’s correction for multiple testing. Considering the number of examined situations, the level of significance after Bonferroni’s correction was determined at *p* = 0.017.

The assessment of a subjectively perceived level of difficulty was performed using the Wilcoxon pair test. The assessment concerned a comparison of the subjectively perceived level of simplicity/difficulty of GI before and after the actual gait in both sitting and standing postures.

## 3. Results

The study results demonstrated significant decreases in muscle activity for the left TA in GI-2 compared with the non-GI condition (*p* = 0.004) and compared with the GI-1 (*p* = 0.009) in the sitting position. The right TA had significantly lower muscle activity in the GI-2 condition in comparison to the GI-1 in both the sitting (*p* = 0.008) and standing (*p* = 0.01) positions. No significant differences were found for the GM. The EMGrms values for the individual muscles and situations that were tested are listed in Table 1.

The subjective perception of the simplicity of GI-1 was rated lower by participants in both sitting (2.53 ± 0.88) and standing positions (2.88 ± 0.87) in comparison to GI-2 in sitting (3.16 ± 0.72) and standing (3.41 ± 0.76) positions. In our experiment, the subjective perception of GI simplicity significantly improved immediately after the real walking in both sitting (*p <* 0.001) and standing (*p <* 0.001) postures.

## 4. Discussion

In the current study, we evaluated the immediate effect of GI on lower-limb muscle activity with respect to the default posture (sitting and standing) in order to explore mechanisms that might contribute to the overall neural activity behind imagining walking. The GI immediately following the non-GI condition, in both sitting and standing default postures, did not lead to any significant changes in muscle activity in this study. The conclusion that GI does not lead to changes in lower-limb muscle activity was also previously reached in study concerning brain activity during GI tasks [16].

Significant changes in muscle activity were found when the non-imagery condition was compared to imagination after gait execution for both the left and right TA in the sitting position and for right TA in the standing position. In both positions, we found a decrease in sEMG activity. After gait execution, participants even reported an improved subjective perception of imagined gait. According to our results, it seems that only the immediately preceding perceptual sensorimotor experience (in our case, actually walking the corridor) resulted in a change in muscle activity during GI and that this experience even improved the subjective inner perception of GI.

### 4.1. Electromyographic Activity during Motor Imagery

Studies focused particularly on central nervous system activity point out the facilitatory effect of motor imagery on excitation at the cortical and subcortical level [9,12,13,14]. The truth is that motor command during imagination of motion must be somehow inhibited to avoid overt movement execution [3,29]. In cases where an increase in sEMG activity is present as a result of motor imagery, the subliminal intensity of the signal may be observed [3,19,29] and its intensity and pattern are not comparable to that present during the execution of real motion.

The sEMG signal reflects the muscle activity, which is determined by the summation of the final motor unit action potentials and their time-course [51,52]. The motor unit (all muscle fibers innervated by one alpha motoneuron’s axons) serves as the transducer of both descending and synaptic sensory inputs transmitted to the motoneuron pools, which are further converted into mechanical muscle output [52,53]. Even the motoneuron intercellular properties may be modulated in different ways [53] from the traditional point of view. I, in case the sum of the synaptic inputs rises above the motoneuron’s threshold for firing, action potentials are generated at a rate proportional to the amplitude of input [53].

The proper mechanisms of muscle activity facilitation or inhibition during motor imagery are not known at present. Processes that inhibit motor action might originate at the cortical, subcortical or spinal level [3,18,29,54]. It is presumed that a strong inhibitory mechanism exists at the spinal level, where the activity of motoneurons (innervating muscles potentially involved in imagined motion execution) is inhibited at the pre-synaptic level [18,49].

It appears that the effect of movement imagery on muscle activity is more pronounced within complex movements when the imagined motion is realized from the first-person perspective, and with proprioceptive feedback corresponding to imagined movement [20,27,28,29], as all these conditions provide enhanced facilitatory inputs to the motoneuron pool. Guillot et al. [29] pointed out that although the sEMG activity is subliminal, the electromyographic signal magnitude correlates to the mental effort that is required.

### 4.2. Factors That May Contribute to the Character of Electromyographic Activity When Imagining

#### 4.2.1. Imagination of Gait

It is possible that gait imagery influences the sEMG activity of target muscles less in comparison to imagery of upper limb motor tasks [23,24] and also in comparison to the imagery of non-gait foot tasks [18,26] as, in these conditions, increased electromyographic activity was found. As we found no effect of the first GI condition on distal lower-limb muscle activity, it may be suggested that this could be, among other factors, due to the automacy of the gait, which is most probably significantly modulated by afferent sensory feedback, during which cortical input might be suppressed [7,14,23,26,39], especially in situations when no additional demands on gait imagination are required.

McCrea [45] previously suggested the importance of proprioceptive feedback for the regulation of muscle activity as a result of locomotor-dependent reflexes during mammalian gait. Feedback from ankle extensor proprioceptors, which induce the reflexive locomotor-dependent activity, contributes to the activity of ankle extensor muscles (medial gastrocnemius) during real walking. These results were further supported by Mayer et al. [46], who proved that feedback from the muscle spindles of ankle extensor muscles is the main source for the modulation of muscle activity strength and speed during gait, and by Dietz [22], who pointed out that leg extensor activity during gait is load-dependent. It was previously demonstrated that spinal-cord-injured humans, who lack supraspinal input below the level of their lesion, can generate oscillating patterns of lower-limb muscle activity with respect to the phasic peripheral sensory information as the lower-limb load changes during assisted gait on treadmill [39].

It is possible that stereotypic gait imagery tasks without any challenge, which were tested in our experiment, do not activate supraspinal motor regions and, subsequently, descendent spinal pathways that could provide a facilitatory neural drive when imagining gait that is sufficient to evoke muscle activity. Furthermore, it seems that the sitting position, in which walking is impossible, might instead have an inhibitory effect on muscle activity during GI, as previously proven [37].

#### 4.2.2. Default Posture during Gait Imagination

It was previously suggested that a posture congruent with the imagined task facilitates corticomotor, corticospinal and muscle–spinal reflex loop excitability within imagery tasks [9,12,18]. Therefore, it was hypothesized that a standing posture would modulate muscle activity within standing to a greater extent in comparison to sitting. Our previous study [37] showed that standing posture facilitated, during imagination of rhythmic gait, muscle activity of proximal lower-limb muscles but not the activity of distal leg muscles. The results of this study further confirm the previously published results [37], namely that the decrease in sEMG activity of distal leg muscles was more evident in the sitting posture in comparison to standing posture. These finding were significant for the GI-2 tested situation.

A standing posture, which is congruent with real walking, provides more appropriate somatosensory feedback (tactile, proprioceptive and visual) compared to postures incongruent with gait execution, such as sitting or lying. Based on the previous findings concerning the effect of sensory information perception from the periphery during imagination [28,29,30,31,41,42], it may be suggested that sensory feedback reflecting real conditions for motion execution during motor imagery increases corticospinal excitability [9,12,30] and strengthens the synaptic sensory inputs transmitted to the motoneuron pool, which further facilitate muscle activity. In the standing posture, the facilitatory sensory input may be provided via muscle spindle in afferent fibers, among other factors, as the increased tension of lower-limb muscles is present during standing in comparison to sitting or lying. Furthermore, a standing posture, compared to a sitting posture, reduces Ib inhibition, which is also connected to foot loading [55]. Therefore, it might be supposed that the mechanisms causing the sEMG decrease, which was present mostly in the sitting position during GI tasks in this study, and even in our previous study [37], occur mostly at the spinal level. This assumption may be supported by previous studies demonstrating increased excitability of the stretch reflexes or increased motor-evoked potentials in the standing position in comparison to sitting or lying [31,42], or in situations when the standing posture was accomplished passively using gait orthosis [42].

When imagining gait in a position in which walking is impossible, the inhibitory effects might dominate over possible facilitatory effects on muscle activity [37]. It is probable that the distal lower-limb motor neurons during stereotypical rhythmic gait imagery tasks in the sitting position do not receive appropriate facilitatory somatosensory input to evoke muscle activity.

The reason that muscle activity changes in the TA are more manifested in the sitting position may be also because it is more of a resting position, where there is no need to simultaneously generate the necessary muscle activity to maintain an upright standing posture. Authors focusing on the application of motor imagery in sports even mention the sitting posture as a more relaxing position, in which it is easier to concentrate on the imagery [56].

#### 4.2.3. Previous Sensorimotor Experience with Real Walking

The decrease in sEMG amplitude as a consequence of gait imagination was already proven for GI, GI in combination with observation and GI after gait execution [37]. As was stated previously, the decrease in muscle activity may reflect, to some extent, the inhibitory effect of motor imagery on motion execution [37,57], although other factors may come into play here.

The fact is that in our study, muscle activity decreases were present just after gait execution in the default sitting posture. Muscle activity decreases were previously described for GI after previous sensorimotor experience with real gait [37] and even for upper-limb tasks as an immediate consequence of previous practice of the task in imagination [58]. The present study demonstrated that significant changes in muscle activity occurred after gait execution and this situation was also significantly easier for the tested subjects to imagine, i.e., situations where the actual somatosensory experience with the imagined movement had already created perceptual footprints in the motor memory led to improved subjective perception of gait imagination and significant decrease in muscle activity. Particularly good motor imagery, based on repeated sensorimotor experience with the trained movement, is demonstrably linked to the attainment of better movement results, e.g., in athletes [59] who consciously and regularly use motor imagery as part of their training [60]. In addition, it was previously stated that motor imagery immediately following the actual realization of the movement facilitates subsequent motor task execution [33,61] as a result of neural adaptations [33], which might further potentiate motor learning processes [5,61]. Previous sensorimotor experience has the potential to refine the internal model of motion [7,46].

Significant changes, understood here as muscle activity decreases, were only observed in the TA, rather than in the GM. Both muscles contribute significantly to gait execution—the TA contributes to dorsiflexion and the slight supination of the foot, especially the swing phase of the gait cycle [62] and the GM participates in the push-off phase [63]. However, from the perspective of surface electromyographic records, the muscle fibers of TA are organized parallelly and the muscle belly is more clearly definable in comparison to the GM, which has a pennate structure with wider muscle belly, so the bipolar sEMG might have limitations to observe discrete changes resulting from gait imagination.

### 4.3. Study Limitations

The results of our study are limited to young, healthy adults with very good motor imagery skills. A limit may be even considered due to the fact that just distal leg muscles were evaluated. Further research is needed to observe the effects of motor imagery in different populations of patients with movement disorders. Another limitation of this study, like other studies focused on this area, is the fact that it is generally very difficult to quantify the quality of imagery or hidden cognitive strategies and undesirable imagery.

### 4.4. Implications for Clinical Practice

Our findings might contribute to the understanding of neural mechanisms of GI and may also provide further insight into the mechanisms beyond motor recovery as a consequence of training in imagination. From the perspective of practical utilization of gait imagery in rehabilitation settings, this study supports the necessity of previous sensorimotor experience with imagined movement and highlights the importance of the default posture during imagination as both these factors have potential to modulate muscle activity.

## 5. Conclusions

The results of our study show that GI influenced the lower-limb sEMG activity in cases when GI immediately followed previous sensorimotor experience of real gait. GI performed immediately after real walking led to a decrease in TA activity, with this occurring bilaterally in the sitting position and unilaterally in the standing posture. The results prove that even previous experience with imagined movement facilitates the subjective perception of GI. These findings have potential to contribute to the understanding of neural mechanisms beyond GI.

## Figures and Tables

**Table 1:** Surface electromyographic activity values (µV) for the individual muscles and situations that were tested.

		Non-Gait Imagery Condition (non-GI)	Gait Imagery before Walking (GI-1)	Gait Imagery after Walking (GI-2)	Friedman’s ANOVA	Wilcoxon Post hoc Test		
						non-GI vs. GI-1	non-GI vs. GI-2	GI-1 vs. GI-2
		Mean (SD)	Mean (SD)	Mean (SD)	*p*	*p*	*p*	*p*
SITTING	Right TA	5.85 (5.33)	5.96 (5.41)	4.67 (2.44)	0.024	0.775	0.029	0.008 *
	Left TA	5.35 (3.29)	5.38 (3.69)	4.36 (1.98)	0.017	0.948	0.004 *	0.009 *
	Right GM	4.39 (2.56)	4.47 (3.3)	3.98 (2.35)	0.002	0.836	0.038	0.066
	Left GM	4.22 (2.57)	4.26 (2.94)	3.76 (1.85)	0.048	0.824	0.124	0.118
STANDING	Right TA	5.44 (3.42)	6.06 (5.46)	4.54 (2.87)	0.042	0.966	0.059	0.01 *
	Left TA	5.56 (2.46)	6.47 (7.3)	4.73 (2.32)	0.249	0.726	0.085	0.413
	Right GM	6.89 (4.1)	7.69 (5.93)	7.46 (4.67)	0.663	0.13	0.431	0.844
	Left GM	7.71 (4.32)	8.74 (4.96)	7.85 (5.62)	0.134	0.379	0.342	0.042

Legend: TA—tibialis anterior, GM—gastrocnemius medialis, non-GI—non-gait imagery condition, GI-1—Gait imagery before walking, GI-2—gait imagery after walking, vs.—versus, SD—standard deviation, *—level of significant difference after Bonferroni corrections (*p* < 0.017).

## Data Availability

The data presented in this study are available on request from the corresponding author.
